# Finite Element Analysis of the Size Effect on Ceramic Strength

**DOI:** 10.3390/ma12182885

**Published:** 2019-09-06

**Authors:** Kyohei Takeo, Yuya Aoki, Toshio Osada, Wataru Nakao, Shingo Ozaki

**Affiliations:** 1 Division of Systems Research, Faculty of Engineering, Yokohama National University, Yokohama 240-8501, Japan (Y.A.) (W.N.) (S.O.); 2 Research center for Structural Materials, National Institute for Materials Science, Tsukuba 305-0047, Japan

**Keywords:** FEM, damage model, Weibull distribution, flaw, effective volume

## Abstract

The most prominent effect of the weakest link theory, which is used to derive the Weibull statistics of ceramic strength, is the size effect. In this study, we analyze the size effect on ceramic strength using the finite element analysis (FEA) methodology previously proposed by the authors. In the FEA methodology, the data of the microstructure distribution (i.e., relative density, size, and aspect ratio of the pore and the grain size) are considered as input parameters of a continuum damage model via a fracture mechanical model. Specifically, we examine five sizes of rectangular specimens under three types of loading conditions. Then, we simulate the fracture stresses of sets of 30 specimens under each size and loading condition and obtain the relationship between the scale parameter and effective volume using the Weibull distribution. The results suggest that the proposed FEA methodology can be applied to the analysis of the fracture probability of ceramics, including the size effect.

## 1. Introduction

Ceramic matrix composites are lightweight and exhibit excellent heat resistance and oxidation resistance. Hence, they are utilized in various high-temperature members/components. In recent years, attempts have even been made to implement ceramics in structural components that require higher safety, including next-generation aircraft engine components. However, as ceramics are brittle materials and exhibit a probabilistic fracture behavior, they are still not highly reliable.

The probabilistic fracture behavior of ceramics is due to brittle fractures originating from inherent “defects (flaws, scratches)” [1,2,3,4,5,6]. Ceramics contain various defects distributed inside the bulk material and on the surface. These defects are caused by sintering processes or subsequent processing, and their distribution characteristics may be different even for products of the same lot. For this reason, the scatter in fracture strength emerges for each specimen or structural components. In addition, ceramic strength depends on size [7]. This is known as the “size effect” and is a direct consequence of statistical distributions and the weakest link theory. The size effect of strength is an important and relevant consequence of the stochastic behavior of the strength of brittle materials [2,3,4,5,6,7,8]. Therefore, it is difficult to follow directly the procedure from “standardized material tests” to “structure design at actual size,” which is used for general metal-like materials.

Accordingly, statistical methods based on the Weibull distribution have been adopted until now for the strength evaluation of ceramics as brittle materials [2,3,4,5,6,7]. The Weibull distribution is generally understood as the following theory: While pulling a plurality of connected chains, entire chains can be destroyed by destroying the weakest link (referred to as the weakest link theory). The most prominent effect of the weakest link theory is the size effect on strength, which results from the simple fact that it is more probable to find a larger and hence weaker pore critically located in a large specimen than in a small specimen under similar loading conditions. Thus, it is possible to grasp the relationship between the volume and strength of a component using the Weibull distribution, in a certain range. However, numerous tests are required to obtain the Weibull distribution, which are expensive and time consuming. Therefore, a numerical method of predicting the scatter in strength, which reflects the distribution of the defects generated in materials during manufacturing processes, is required.

Several methods that consider the stochastic distribution of flaws and use Monte Carlo simulations, etc., have been reported to predict the scatter in the strength of ceramics [4,5,8,9,10]. Furthermore, a reliability evaluation method that combines the detailed structural analysis result obtained using the finite element method (FEM) and the Weibull statistical results of a standardized specimen has been proposed to examine the case where an arbitrary load acts on a component with an arbitrary shape [11,12,13,14].

Meanwhile, the authors proposed a finite element analysis (FEA) methodology that predicts the scatter in ceramic strength based on microstructure data (relative density, defect distribution, grain size distribution, etc.) [15]. Specifically, the information of the microstructure distribution obtained through image observation is represented by various probability density functions. This allows for strength analysis based on the FEM by indirectly reflecting the parameters of a continuum damage model via a fracture mechanics model. In addition, it is possible to reproduce the stochastic distribution of a microstructure in a number of analysis targets (for example, tens to hundreds of specimens) by assuming the same lot using random numbers. As a result, we succeeded in directly creating a Weibull distribution from the results of FEA, in which different fracture strengths were predicted for each analysis target.

In this study, we show that not only the scatter in the strength of same-size specimens, but also the size effect can be considered by the previously proposed FEA methodology [15]. Ceramics have different material strengths depending on the test specimen size and test method by size dependency. Therefore, it is important to be able to evaluate the size dependence of ceramics by the proposed FEA methodology [15]. Specifically, we prepared four kinds of finite element (FE) models and analyzed them under three kinds of loading conditions, that is three-point bending, four-point bending, and simple tension. We analyzed *N* = 30 specimens under each condition. Then, we created Weibull distributions and investigated the relationship between fracture strength and effective volume. Herein, the effective volume of a component corresponds to the volume of a hypothetical tensile specimen loaded with the maximum equivalent stress amplitude of the component and having the same failure probability.

The ceramic material we examined was Al_2_O_3_/30 vol% SiC, which is the same as that used in the previous study [15]. The processing defects on its surfaces are repaired in advance through heat treatment because the Al_2_O_3_/30 vol% SiC particle composite has a self-healing property [16,17,18,19]. Thus, it is possible to consider only the fractures that start from internal flaws [16,17].

## 2. Constitutive Model

### 2.1. Isotropic Damage Model

**Figure 1:**
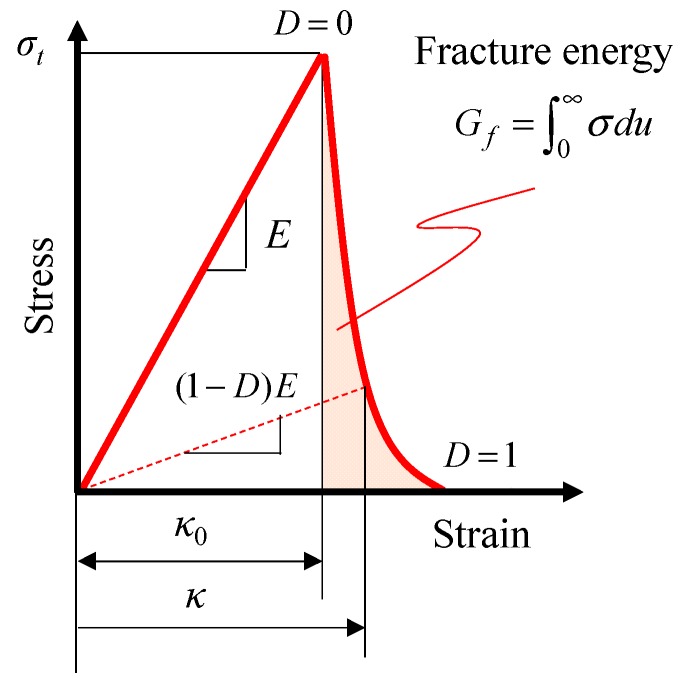
Schematic diagram of the stress–strain relationship in the isotropic damage model.

The stress–strain relationship based on the isotropic damage model adopted in this study is expressed as follows [15,20,21]:

(1) σ=(1−D)c:ε

where σ is the Cauchy stress tensor, ε is the small strain tensor, c is the fourth-order hypo-elastic coefficient tensor, *D* is the damage variable, and *D* = 0 and *D* = 1 correspond to the non-damaged and perfectly-damaged states, respectively. Figure 1 shows the schematic diagram of the response of Equation (1).

The damage variable *D* is a function of the maximum value of the equivalent strain κ, as given as follows:

(2) $$D(\kappa )=1-\frac{\kappa_{0}}{\kappa }\exp\left\{-\frac{E\kappa_{0}h_{e}}{G_{f}}(\kappa -\kappa_{0})\right\},$$

where $\kappa_{0}$ is the equivalent strain at damage initiation; $h_{e}$ is the characteristic length corresponding to the length of the finite element in the FEA; *E* is Young’s modulus; and $G_{f}$ is the fracture energy. The following equivalent strain *κ* according to the modified von Mises model was adopted as an internal state variable of damage variable *D*:

(3) $$\kappa =\frac{k-1}{2k(1-2\nu )}I_{1}+\frac{1}{2k}\sqrt{{\left(\frac{k-1}{1-2\nu }I_{1}\right)}^{2}+\frac{12k}{{\left(1+\nu \right)}^{2}}J_{2}},$$

where *ν* is Poisson’s ratio, *k* is the ratio between tensile and compressive strengths, *I*_1_ is the first invariant of the strain tensor ε, and *J*_2_ is the second invariant of deviatoric strain tensor $\varepsilon^{*}\;(=\varepsilon -tr\varepsilon /3)$.

**Figure 2:**
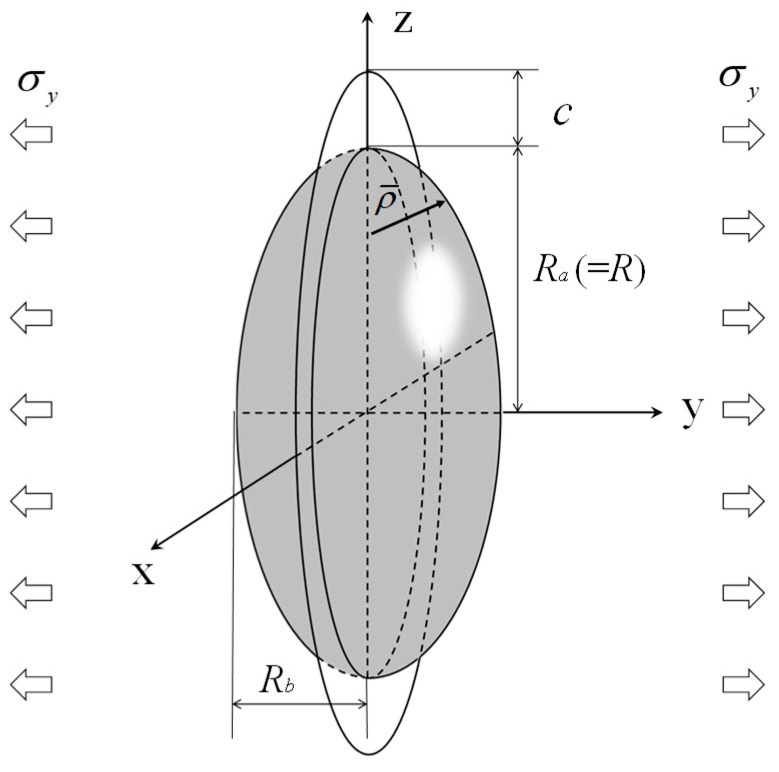
Schematic of the circumferential circular crack emanating from an ellipsoidal pore. The radius in the y-direction is the same as *R*.

In this study, fracture stress *σ_t_* was estimated based on linear fracture mechanics, as follows:

(4) $$\sigma_{t}=\frac{K_{\mathrm{IC}}}{F}\frac{1}{\sqrt{\pi c}},$$

where *K*_IC_ is fracture toughness and *c* is the initial crack length. *F* is the geometric factor obtained from the shape of an oval spherical pore (rotational spheroidal pore) and the initial crack length, as shown Figure 2. Here, *R_a_* = *R* is the major axis radius and *R_b_* is the minor axis radius. The geometric factor for a circumferential crack emanating from an oval spherical pore under tension (Figure 2) is summarized as follows [15,22,23]:

(5) $$F=\{\begin{array}{ll} F_{1} & \mathrm{for}\;c/\bar{\rho }\leq 1,\\ \max(F_{1},\;F_{2}) & \mathrm{for}\;c/\bar{\rho }>1. \end{array}$$

Here,

(6) $$F_{1}=1.125K_{t}\left[\frac{1}{3}+\frac{1}{6}\left\{\frac{1}{{\left(1+\lambda \right)}^{2}}+\frac{3}{{\left(1+\lambda \right)}^{4}}\right\}\right]\left(1+0.2238\lambda -0.1643\lambda^{2}\right),$$

(7) $$F_{2}=\frac{2}{\pi }\sqrt{\frac{R+c}{c}}.$$

$\bar{\rho }$ is the notch root radius, and $\lambda =c/\bar{\rho }$. Note that the initial crack length, *c*, corresponds to grain size, referring to the previous study [9,24,25,26,27,28].

The fracture energy used in the damage model is given as follows:

(8) $$G_{f}=\frac{K_{\mathrm{IC}}^{2}}{E}\left(1-\nu^{2}\right).$$

Herein, we assumed Mode I cracking in a plane strain condition. In addition, the equivalent strain at damage initiation ($\kappa_{0}$) was set by assuming a uniaxial tensile fracture, as follows:

(9) $$\kappa_{0}=\sigma_{t}\frac{\left(1+\nu )(1-2\nu \right)}{E(1-\nu )},$$

where *E* is Young’s modulus. The fracture strength of ceramics depends on the basic material properties and microscopic structure such as defect size and grain size, because the equivalent strain of each element is damaged when it reaches the damage initiation strain *κ*_0_.

Concrete equations, such as stress concentration factor *K_t_*, were provided in Ozaki et al. [15].

### 2.2. Parameter Evaluation Based on Microstructure

In ceramics, microstructure data, such as pore size and grain size, are stochastically distributed. Therefore, Ozaki et al. [15] proposed an FEA methodology that can evaluate the scatter in ceramic strength by varying the microstructure data for each element using probability density functions and random numbers. In this study, the stochastic distribution of a microstructure was expressed in the same manner.

The distributions of relative density, the aspect ratio of a pore, and grain size were defined by the probability density functions of the half-normal distribution, normal distribution, and log-normal distribution, respectively, as in the previous paper [15]. However, the probability density function for the pore size distribution was newly modified by an inverse power law, even though the half-normal distribution or log-normal distribution was adopted in the previous study. The power law distribution is expressed as shown in the following equation (*b_R_* ≠ 1):

(10) $$F\left(R\right)=\{\begin{array}{cc} 0 & R\leq R_{\min},\\ \frac{R_{\min}^{1-b_{R}}-R^{1-b_{R}}}{R_{\min}^{1-b_{R}}} & R_{\min}\leq R, \end{array}$$

where *F*(*R*) is a cumulative distribution function and *R*_min_ is the minimum value of the pore radius. Moreover, *b_R_* is the parameter used to determine the shape of the power law curve (power index). Note that the minimum value of the pore radius depends on the size of one finite element. Incidentally, for the case that one must consider the largest threshold of pore size, the different power law distribution can be adopted (see the Appendix A).

## 3. FE Model and Boundary Condition

**Figure 3:**
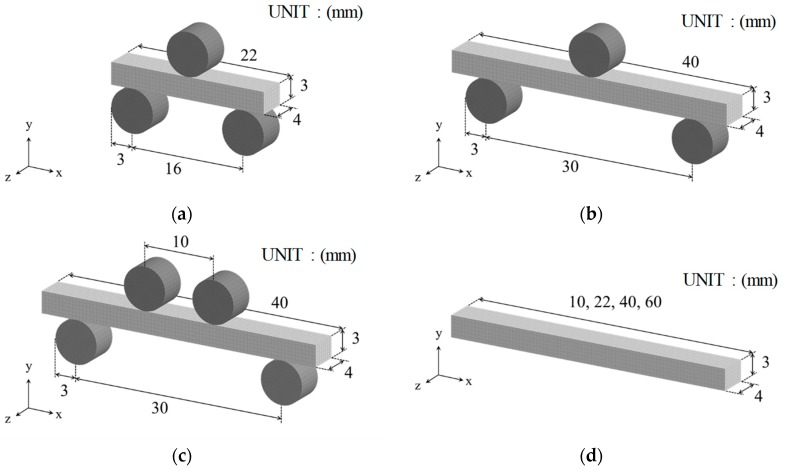
FE models: (**a**) three-point bending Test A (span is 16 mm); (**b**) three-point bending Test B (span is 30 mm); (**c**) four-point bending test; (**d**) tensile test.

Figure 3 shows the FE analysis models used in this study. Figure 3a,b shows the three-point bending tests with different sizes of specimens, and Figure 3c shows the four-point bending test, while Figure 3d shows the tensile test. The thickness and width of the specimens were 3 and 4 mm, respectively. These were the same for all models. In addition, four values (10, 22, 40, and 60 mm) of the lengths of specimens were considered for the tensile test (Figure 3d) to simply change the effective volume. The jigs of the three-point bending test and four-point bending test were set as rigid bodies. In the calculations of the bending tests, a constant forced velocity (constant crosshead speed of 5 mm/s) in the vertical direction was imposed on the upper jig, while the displacements of the lower jigs were fixed. A friction coefficient of 0.3 was set at the contact boundary between the specimen and jigs. In the calculations of the tensile test, tensile load was applied by completely fixing one end of the specimen and applying forced displacement (constant velocity of 3 mm/s) to the other end. Note that the major axis orientation of pores corresponds to the y-axis direction in Figure 3.

In this study, the commercial FEM software package LS−DYNA (r7.1.2, Livermore Software Technology Corporation, California, CA, America) and its material user subroutine, umat XX [29], were utilized. A dynamic explicit method was adopted to perform a numerical integration in the time domain. All specimens shown in Figure 3 were discretized by an eight-node solid element with dimensions of 0.5 mm × 0.5 mm × 0.5 mm. Moreover, one-point Gaussian integration was adopted.

The material properties and the condition of the microstructure distribution are shown in Table 1 and Table 2, respectively. In the present FEA methodology, the statistical dispersion of the material parameters for each element was set based on the material properties given in Table 1 and the average and standard deviation of the microstructure distribution given in Table 2.

**Figure 4:**
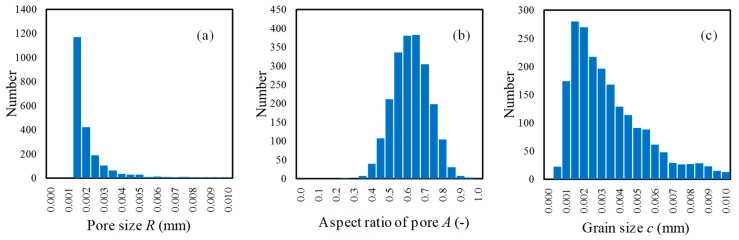
Histogram of the microstructure data of the elements obtained for an arbitrary specimen of the four-point bending test (length 40 mm) as shown in Figure 3c. The distribution of the microstructure is defined as given in Table 2: (**a**) pore size *R*; (**b**) aspect ratio *A*; and (**c**) grain size *c* (initial crack length).

Figure 4 shows an example of the histograms of microstructure data obtained under the conditions listed in Table 2. Here, the class (abscissa) and frequency (ordinate) of the histograms correspond to the values of each element and the number of elements, respectively. The data in the histograms were obtained for an arbitrary specimen with a length of 40 mm of the four-point bending test model. Based on the statistically-distributed microstructure data and using Equations (4), (8) and (9), the fracture stress *σ_t_*, fracture energy *G_f_*, and strain *κ*_0_ at damage initiation were evaluated for each element (Gaussian point). The above-mentioned parameter setting operation for the damage model was performed only at the Time 0 step.

Figure 4a shows that the existence probability of the likelihood of a pore being present within a specified amount of volume decreases as pore size increases, according to the inverse power law distribution. The minimum value of the histogram corresponds to the maximum pore size that would exist in the volume per element. Note that, even though pores of several sizes were found in one element, we neglected the presence of micro pores and the interference between pores (the overlap of stress fields). Moreover, we only considered the largest pore, which might become a fracture origin in each element.

## 4. Results and Discussion

**Figure 5:**
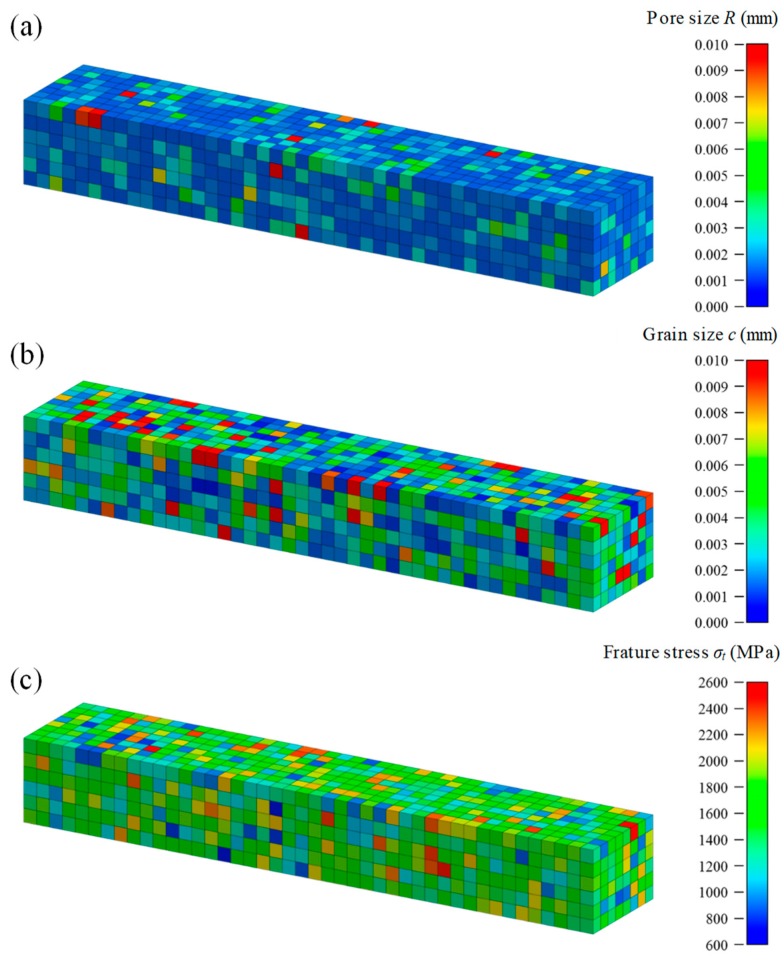
Distributions of microstructure data in three specimens arbitrarily extracted from four-point bending test models. The contour map shows: (**a**) pore size *R*; (**b**) grain size *c*; and (**c**) fracture stress *σ_t_*.

Figure 5a–c shows the contour maps of pore size, grain size, and fracture stress, respectively. It can be confirmed from the figure that fracture stress is distributed stochastically based on the different pore size and grain size of each element. Moreover, we confirmed that each specimen represents a different distribution by random numbers.

**Figure 6:**
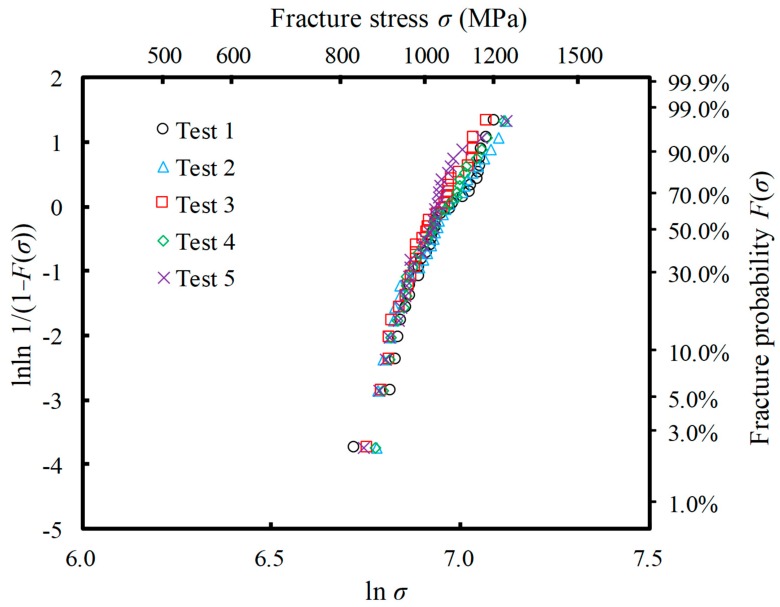
Weibull plots of fracture strength distributions obtained by the virtual test. This was conducted five times using FEA of the four-point bending, in which *N* = 30 specimens were used. The microstructure distribution condition used is given in Table 2.

Figure 6 shows the Weibull distribution obtained by the FEA of the four-point bending test. The graph includes the results of five tests. The number of specimens in each dataset was set as *N* = 30. Even if the same microstructure distribution condition shown in Table 2 were used for each specimen, the parameters of the damage model set for each element were random, and thus, different Weibull distributions were obtained. However, note that the Weibull modulus and scale parameter were almost the same in the results of the five tests because we assumed the same lots of the Al_2_O_3_/30 vol% SiC particulate composite.

**Figure 7:**
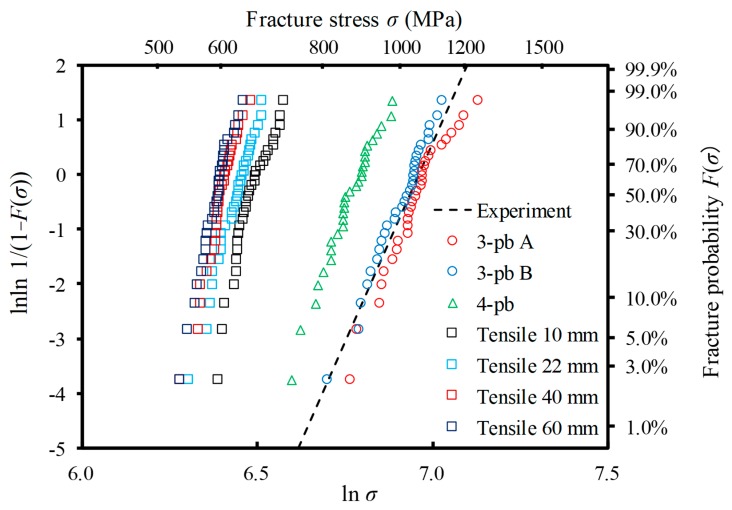
Numerically-obtained Weibull distributions for seven tests. For each test, *N* = 30 specimens were used. The dashed line is the experimentally-obtained result of the Al_2_O_3_/30 vol% SiC particle composite (*m* = 14.5 and *β* = 1055 MPa) [16].

Figure 7 shows the Weibull distribution obtained by each analysis model shown in Figure 3. Here, the number of specimens in each dataset was set to *N* = 30. For the sake of reference, the experimental result [16] of the three-point bending test with the same specimen size as the three-point bending Test A is shown as represented by the broken line. As the effective volume increased, the scatter of strength decreased, and the Weibull modulus *m* increased, in the range of the examined condition. Moreover, it can also be confirmed that the scale parameter *β* representing the mean strength decreased with the increase in effective volume. It is thought that these tendencies depend on the distribution characteristics of the microstructure. The present methodology can analyze the fact that the fracture of ceramics is caused by the probability of the existence of large flaws in the part subjected to a tensile stress. Hence, the predicted strength depends on the effective volume. Table 3 lists the Weibull modulus *m* and the scale parameter *β* obtained by FEA for each test model.

**Figure 8:**
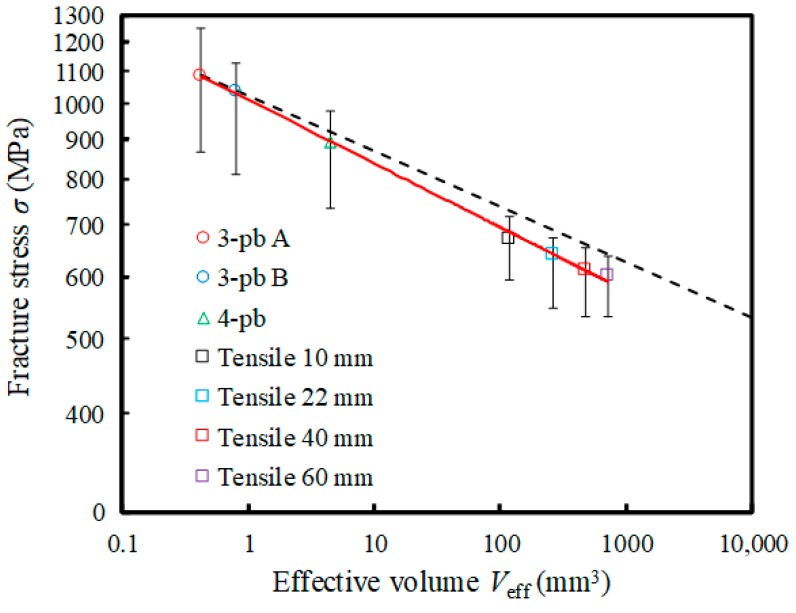
Relationship between the scale parameter *β* and the effective volume *V_eff_* obtained by FEA. The dashed line shows the extrapolation line using the Weibull modulus *m*_3A_ of the three-point bending test (Figure 3a).

Figure 8 shows the relationship between the mean strength (scale parameter *β*) and effective volume *V_eff_* in log-log scale. Here, in the bending tests, the effective volumes of each specimen were evaluated using the Weibull modulus *m*_3A_ of three-point bending Test A, as shown in Table 3. Each error bar on the plots indicates the maximum strength (fracture probability: *P*(*σ*) = 97.7%) and minimum strength (fracture probability: *P*(*σ*) = 2.3%). As in previous reports [4,5,6,7,8], the scale parameter decreased with increasing effective volume in the logarithmic scales. The solid line in the graph indicates the approximate line obtained by the least squares method. It was confirmed that the results obtained by the present FEA exhibited a trend similar to that predicted by the Weibull theory.

In general, in ceramics, if the effective volume and the scale parameter obtained by tests are *V*_0_ and *σ*_0_, respectively, the fracture strength *σ_t_* in different tests having effective volume *V_eff_* is regarded as following the relation:

(11) $$\sigma_{t}{}^{m}V_{eff}=\sigma_{0}^{m}V_{0}.$$

Herein, the Weibull modulus *m* was assumed to be equal in certain size ranges in that relation. The broken line in Figure 8 shows the estimated mean strength using the Weibull modulus *m*_3A_ of three-point bending Test A, which was the smallest specimen. From the comparison of the broken line with the plots, it can be seen that the conventional estimation based on Equation (11) predicted dangerous side strength. In particular, when the effective volume became 100-times or more, the estimated strength became a higher value. In the case of the tensile test, it is rational to think that the existence probability of large pores drastically increases, and the scatter of strength becomes small as the effective volume increases. It is thought that the present FEA methodology can deal with such situations.

Meanwhile, the present FEA methodology can naturally predict both the Weibull modulus and mean strength for specimens having arbitrary sizes because an effective volume does not need to be evaluated in advance. This enables the evaluation of the safety side strength (see the approximate solid line in the graph in Figure 8). Moreover, the present methodology can predict the lower limit of strength from the fracture probability (e.g., *P*(*σ*) = 2.3% in the case of *N* = 30), which is the lower limit of the error bar, and enables further reliability evaluation. Therefore, even with brittle materials, it is possible to design components/members in accordance with safety requirements considering the “lower limit of strength.” It is also thought that the present methodology can be used for optimization of the microstructure distribution, sintering processes, and subsequent processing, according to the shapes of components/members.

## 5. Conclusions

In this study, we investigated the size effect of ceramics by using the FEA methodology [15], which can predict the scatter of the strength of ceramics based on microstructure information. Four kinds of specimen sizes were prepared, and three kinds of loading tests were carried out. We then analyzed *N* = 30 specimens for each condition to create Weibull distributions. The results in the examined condition showed that the Weibull modulus increased and the scatter of strength decreased as the effective volume increased, since the probability of existence of large pores (defects) increased. Furthermore, it was also shown that the scale parameter decreased with increases in the effective volume in log-log scale, and the data obtained by FEA followed the trend of the Weibull theory. It was possible to analyze the size effect on strength, which is an important and relevant consequence of the statistical behavior of brittle ceramics, by using the present FEA methodology.

Moreover, unlike the conventional method, in which the Weibull modulus is assumed to be a constant value, the present methodology can enable us to evaluate the safety side strength because it can naturally analyze the fracture behavior under any given shape and boundary conditions.

## Figures and Tables

**Table 1:** Basic material parameters. *k* = 10 means the tensile strength is ten-times the compressive strength.

*E*_100_ (GPa)	*ν*	*K*_IC_ (MPa m^1/2^)	*k*
398	0.21	3.8	10

**Table 2:** Distribution characteristics of the microstructure. The mean value and standard deviation of the half-normal distribution are those before half-folding of the normal distribution.

	**Type of Distribution**	**Mean Value**	**Standard Deviation**
Relative density *ρ*	Half-normal distribution	*μ_ρ_* = 0.99	*σ_ρ_* = 0.0
Aspect ratio *A*	Normal distribution	*μ_A_* = 0.6	*σ_A_* = 0.1
Grain size *c* (mm)	Log-normal distribution	*E_c_* = 3.6×10^−3^	*V_c_* = 9.79×10^−6^
	**Type of Distribution**	**Exponent**	**Minimum Value**
Pore size *R* (mm)	Power law distribution	*b_R_* = 3.0	*R*_min_ = 0.001

**Table 3:** Weibull parameter *m*, scale parameter *β*, and effective volume *V_eff_* corresponding to Figure 7.

	*m*	*β* (MPa)	*V_eff_* (mm^3^)
3-point bending A	14.1	1088.7	0.42
3-point bending B	15.8	1038.9	0.79
4-point bending	16.9	892.8	4.50
Tensile 10 mm	22.5	671.7	120
Tensile 22 mm	23.4	639.7	264
Tensile 40 mm	28.2	612.3	480
Tensile 60 mm	28.7	602.7	720

**Figure A1:**
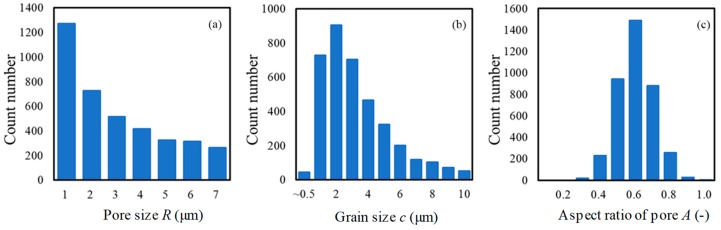
Histogram of the microstructure data of the elements obtained for an arbitrary specimen of the four-point bending test (length 40 mm) as shown in Figure 3. The distribution of the microstructure is defined as given in Table 2, while *μ_R_* = 0.8 and *μ_R_* + 3*σ_R_* = 7.87 for the pore size: (**a**) pore size *R*; (**b**) aspect ratio *A*; and (**c**) grain size *c* (initial crack length).

**Figure A2:**
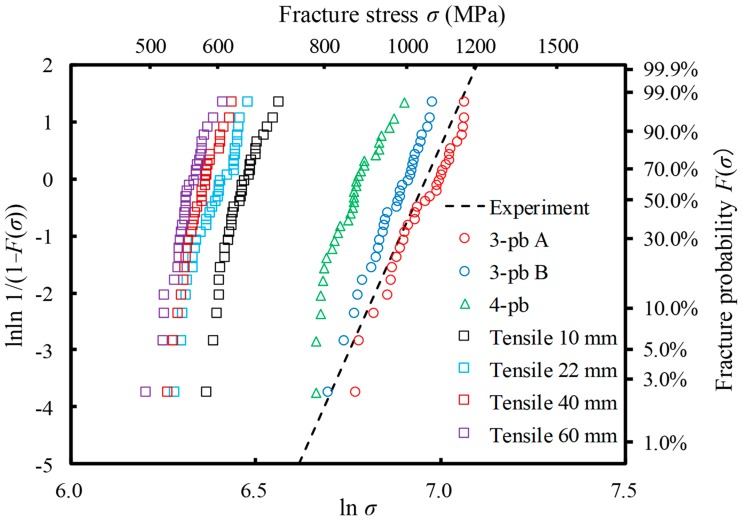
Numerically-obtained Weibull distributions by using Equation (A1). For each test, *N* = 30 specimens were used. The dashed line is the experimentally-obtained result of the Al_2_O_3_/30 vol% SiC particle composite (*m* = 14.5 and *β* = 1055 MPa) [16].

## Appendix A. Pore Distribution with Maximum Value

In this section, we explain the probability density function for the pore size, in which the maximum threshold of size is introduced.

The power law distribution is expressed by translating a uniform random number to a power law random number, as shown in the following equation:

(A1) $$x={\left[F(x)\left\{{\left(\mu_{R}+3\sigma_{R}\right)}^{1-b}-\mu_{R}{}^{1-b}\right\}+\mu_{R}{}^{1-b}\right]}^{\frac{1}{1-b}}.$$

where *x* is a power law random number, *F*(*x*) is a uniform random number, *μ_R_* is the minimum value of the pore radius, and *σ_R_* is the standard deviation, i.e., (*μ_R_* + 3*σ_R_*) is the maximum value of pore radius (corresponding to the 99.73% limit). Moreover, *b* is the parameter used to determine the shape of the power law curve (power index). Note that the minimum and maximum values of the pore radius depend on the size of one finite element and the entire analyzed model (e.g., volume of specimens), respectively.

Figure A1 shows an example of the histograms of microstructure data. Here, the class (abscissa) and frequency (ordinate) of the histograms correspond to the values of elements and the number of elements, respectively. The data in the histograms were obtained for an arbitrary specimen with a length of 40 mm of the four-point bending test model (see Figure 3c).

Figure A1a shows that the existence probability decreased as pore size increased, according to the inverse power law distribution of Equation (A1). The minimum value of the histogram corresponds to the maximum pore size that would exist in the volume per element. Furthermore, the maximum value of the histogram corresponds to the maximum pore size (*μ_R_* + 3*σ_R_*) that would exist in the entire volume of the analyzed model. Therefore, the maximum value of pore size differs according to the size of specimens shown in Figure 3. The maximum pore sizes (*μ_R_* + 3*σ_R_*) of the specimens with lengths of 10, 22, 40, and 60 mm were 6.00, 7.00, 7.87, and 8.52 μm, respectively.

Figure A2 shows the Weibull distribution obtained by each analysis model shown in Figure 3. Here, the number of specimens in each dataset was set to N = 30. For the sake of reference, the experimental result [16] of the three-point bending test with the same specimen size as the three-point bending Test A is shown as represented by the broken line. It showed the same tendency as when the maximum value of the pore was not determined. Therefore, the appropriate analysis results were shown by determining the distribution according to the material.
